# Home visits by community health workers for pregnant mothers and newborns: coverage plateau in Malawi

**DOI:** 10.7189/jogh.09.010808

**Published:** 2019-06

**Authors:** Tanya Guenther, Humphreys Nsona, Regina Makuluni, Mike Chisema, Gomezgani Jenda, Emmanuel Chimbalanga, Salim Sadruddin

**Affiliations:** 1 Abt Associates, Dili, Timor-Leste; 2 IMCI unit, Ministry of Health, Lilongwe, Malawi; 3 District Health Office, Ntcheu, Malawi; 4 Save the Children Malawi, Lilongwe, Malawi;; 5 Management Sciences for Health (MSH), Lilongwe, Malawi; 6 World Health Organization, Geneva, Switzerland; * At the time this paper was first drafted, Ms. Guenther was employed by Save the Children, USA.

## Abstract

**Background:**

Home visits by community health workers (CHWs) during pregnancy and soon after delivery are recommended to improve newborn survival. However, as the roles of CHWs expand, there are concerns regarding the capacity of community health systems to deliver high effective coverage of home visits. The WHO’s Rapid Access Expansion (RAcE) program supported the Malawi Ministry of Health to align their Community-Based Maternal and Newborn Care (CBMNC) package with the latest WHO guidelines and to implement and evaluate the feasibility and coverage of home visits in Ntcheu district.

**Methods:**

A population-based survey of 150 households in Ntcheu district was conducted in July-August 2016 after approximately 10 months of CBMNC implementation. Thirty clusters were selected proportional-to-size using the most recent census. In selected clusters, five households with mothers of children under six months of age were randomly selected for interview. The Health Surveillance Assistants (HSAs) providing community-based services to the same clusters were purposively selected for a structured interview and register review.

**Results:**

Less than one third of pregnant women (30.7%; 95% confidence interval CI = 21.7%-41.5%) received a home visit during pregnancy and only 20.7% (95% CI = 13.0%-29.4%) received the recommended two visits. Coverage of postnatal visits was even lower: 11.4% (95%CI = 6.8%-18.5%) of mothers and newborns received a visit within three days of delivery and 20.7% (95%CI = 12.7%-32.0%) received a visit within the first eight days. Reaching newborns soon after delivery requires timely participation of the family and/or health facility staff to notify the HSA – yet only 42.9% (95% CI = 33.4%-52.9%) of mothers reported that the HSA was informed of the delivery. Coverage of postnatal home visits among those who informed the HSA was significantly higher than among those in which the HSA was not informed (46.7% compared to 1.3%; *P* = 0.00). Most HSAs had the necessary equipment and supplies and were active in CBMNC: 83.9% (95% CI = 70.2%-97.6%) of HSAs had pregnancy home visits and 77.4% (95% CI = 61.8%-93.0%) had postnatal home visits documented in their registers for the previous three months.

**Conclusions:**

We found low coverage of home visits during pregnancy and soon after delivery in a well-supported program delivery environment. Most HSAs were conducting home visits, but not at the level needed to reach high coverage. These findings were similar to previous studies, calling into question the feasibility of the current visitation schedule. It is time to re-align the CBMNC package with what the existing platform can deliver and identify strategies to better support HSAs to implement home visits to those who would benefit most.

In Malawi, 15 000 (42%) of the estimated 36 000 under-five deaths in 2016 occurred in the first month of life [1]. The majority of these deaths are due to preventable and/or treatable causes, namely complications at birth, complications of prematurity, and infection [1]. Close to three-quarters of newborn deaths occur within the first week of delivery and this period is of great risk for mothers as well [2,3]. Prompt health checks for recently delivered women and newborns in the community can facilitate timely identification and management of complications as well as improve uptake of recommended newborn care practices [2-5].

In 2009, the World Health Organization (WHO) and the United Nations International Children's Emergency Fund (UNICEF) released a joint statement encouraging governments in low-income countries to introduce postnatal home visits for newborns to reduce mortality, including at least two visits within the first week after delivery with the first visit occurring within 48 hours of birth [3]. These recommendations were based on several studies in high mortality settings in Bangladesh, India and Pakistan that had shown promising results on a small scale under research conditions [6-8]. Building on the release of the joint statement and subsequent 2013 WHO guidance on postnatal care, countries started developing policies and programs and by 2016 more than 50 countries had a policy promoting home visits for newborns [9,10]. However, experience implementing postnatal home visits within existing government systems at scale has shown major challenges to reaching high coverage of postnatal home visits [5,11,12]. A recent review of postnatal home visits in 11 countries in Africa and Asia that operated programs at scale found that for most countries, coverage of a postnatal home visit within 48 hours was below 10% and no country achieved greater than 20% [11].

Malawi was an early adopter of pregnancy and postnatal home visits as part of its strategy to reduce maternal and newborn mortality. Starting in 2007, the Ministry of Health (MOH), in partnership with Save the Children in Malawi (SC), UNICEF, WHO and other partners, designed and piloted a Community Based Maternal and Newborn Care (CBMNC) package delivered through CHWs referred as Health Surveillance Assistants (HSAs). The HSAs were trained to conduct three home visits during pregnancy (one per trimester) and three postnatal home visits within the first eight days of delivery (Day 1, Day 3 and Day 8). The CBMNC package was piloted in three districts (Chitipa, Dowa and Thyolo) starting in 2008 with support from Saving Newborn Lives (SNL), a project of SC. By 2011, the package was scaled up to 17 districts and 1781 HSAs had been trained in CBMNC with support from UNICEF, WHO, United States Agency for International Development (USAID) and Norwegian Church. Evaluation in the three pilot districts found that 36% of women received one or more home visits during pregnancy and 11% of newborns received a postnatal home visit within the first three days, and that mothers and babies in the richest quintile were more likely to receive home visits [5,13]. Despite the low coverage, an economic analysis indicated that the program would be cost-effective if coverage could be improved by increasing activity levels of HSAs [14]. In 2014, SC Malawi, under the WHO’s integrated Community Case Management (iCCM) Rapid Access Expansion (RAcE) program, supported the Malawi MOH to design, implement and evaluate a revised CBMNC package in Ntcheu district. In this paper, we present an evaluation of the coverage of CHW home visits during pregnancy and postnatal period and explore household and CHW factors associated with coverage of home visits.

## EVALUATION SETTING AND DESIGN

In 2014, the CBMNC package was revised to align with the *WHO Caring for the Sick Newborn* package [15]. The Integrated Management of Childhood Illness (IMCI) unit of the MOH led the consultative process that included members of the Reproductive Heath Directorate of the MOH and other implementing partners, with financial and technical support from WHO and SC Malawi through the RAcE program. Under the revised CBMNC package, the number of home visits during pregnancy was reduced from three to two, while the number of recommended postnatal home visits remained the same (three visits within the first eight days). During pregnancy home visits, HSAs convey messages regarding the importance of antenatal care and refer women to the health facility for antenatal care, preparing for facility delivery, and recognition and care-seeking for danger signs. During postnatal home visits, scheduled for Day 1 (within 24 hours of delivery), Day 3 and Day 8, the HSAs assess the mother and baby for signs of illness and refer them to an appropriate facility, if required, weigh the baby, and help the mother with early and exclusive breastfeeding and keeping the baby warm. Further details on the timing and tasks of each HSA home visit are given in Table S1 in the **Online Supplementary Document**.

### Implementation of CBMNC in Ntcheu district

The revised CBMNC package was introduced in Ntcheu district. Ntcheu district was selected by the MOH as it was an area where the RAcE iCCM program was well-established, had a strong district health office, and was the site for a linked operations research study testing the feasibility of outpatient management of possible serious bacterial infections in infants under two months of age. Ntcheu is located in central Malawi, along the border with Mozambique, and had an estimated population of 588 038 and 24 835 expected births in 2016 [16]. All HSAs in the district were targeted for training in the package. The CBMNC training was six days and delivered under the leadership of the Ntcheu district maternal and newborn coordinator by nationally recognized trainers who had completed a ‘training of trainers’ course. A total of 299 HSAs were trained between September 2015 and May 2016 through 12 sessions with between 24 and 30 participants per session. Most HSAs (85%; 253/299) had completed training by December 2015. Senior HSAs/Cluster Supervisors as well as district staff were expected to supervise HSAs in CBMNC and they were trained on the package together with HSAs. However, focused training on supervision of CBMNC and provision of supervision checklists for CBMNC were not provided to senior HSAs until November 2016 (after the evaluation). The HSAs were equipped with weighing scales, thermometers, respiratory rate timers, counseling cards, referral slips and registers to document home visits, and monthly reporting forms. Overall, 36 of the 38 health facilities in Ntcheu were providing CBMNC services following the training, with the majority having all associated HSAs trained in the package, except for the district hospital catchment areas where about half of the HSAs completed CBMNC training.

#### Data collection methods

A cross-sectional, population-based household survey was conducted in August 2016 after approximately 10 months of implementation to capture coverage of home visits and maternal and newborn care practices in the program area. A survey of HSAs serving the selected household survey clusters was implemented alongside the household survey to help understand HSA background characteristics, activity levels and program support in terms of supervision and supplies and birth notification.

### Sampling

#### Household survey

A target sample size of 144 mothers of infants under six months of age was calculated for the primary outcome of home visit coverage for mothers and newborns, assuming 80% power, 90% confidence interval, a design effect of 2.0, and a coverage value 40%. Two-stage sampling methodology was used in which 30 clusters of five households were selected for a total of 150 households. Census enumeration areas (EAs) were used to define survey clusters and were selected proportional-to-size using the most recent census (2008). The listing of all EAs for Ntcheu district was obtained from the Malawi National Statistics Office (NSO).

Within selected clusters, all households were listed and a screening questionnaire administered to determine ages of all usual members of the household to identify eligible households with one or more caregivers of infants under six months. This age group was selected to ensure potential exposure to the CBMNC intervention during pregnancy and post-delivery. Households in which an adult representative was not home during the initial contact were revisited once before being considered unavailable. Following the listing, ineligible households were removed and five households with an eligible caregiver were randomly selected for interview. Interviewers administered the questionnaire to the eligible caregiver in selected households; in the case where there were more than one eligible caregiver present in a single household, one was randomly selected.

#### HSA survey

The HSAs providing services to the same 30 clusters sampled for the household survey were purposively selected for an interview using the HSA questionnaire. The objective of the HSA survey was to gain a better understanding of the HSAs’ background characteristics, activity levels, and support and supervision to help interpret the results of the household survey. As HSA catchment areas did not align with the census enumeration areas, it was possible that more than one HSA was associated with a given cluster. In such cases, one HSA was randomly selected for interview.

### Data collection

The household questionnaire was developed based on a 2011 household survey to evaluate the earlier CBMNC program implemented under the SNL project, with further updates made based on the latest Demographic and Health Survey (DHS) model women’s questionnaire Phase 7 [17]. The questionnaire included four sections: 1) maternal background characteristics, 2) exposure to CBMNC interventions, 3) antenatal, delivery and newborn care, and 4) sick newborn care. Similarly, the HSA survey questionnaire was developed based on the 2011 SNL evaluation questionnaire for HSAs. The questionnaire captured HSA background characteristics, training and knowledge of newborn health, newborn health materials and supplies (observation-based), activity levels based on HSA report and register review, and supervision. Both questionnaires were translated into Chichewa and back-translated independently into English to check the accuracy of the translation. Questionnaires were pre-tested in Chichewa by NSO during the data collection training and minor modifications were made to finalize the survey tools.

The survey was implemented by NSO, with technical support from ICF International and SC. The CMBNC evaluation in Ntcheu was nested within a larger evaluation of the iCCM program, which was conducted in the four original RAcE districts (Ntcheu, Mzimba North, Dedza and Ntchisi). Data collectors were full-time NSO staff with experience conducting national household surveys. Data collectors and supervisors were trained by NSO for eight days, including two days of field practice. The training covered an overview of the RAcE project, interviewer roles and responsibilities, household and respondent selection, administering informed consent, question-by-question review of the data collection tools, mock interviews and data quality checks.

Data collection was carried out in August 2016 by nine survey teams of three interviewers each, with one team assigned to CBMNC in Ntcheu district. Each team was led by a supervisor trained to monitor and support the data collectors, review completed questionnaires, and oversee household listing and sampling. Technical staff from ICF International provided support for the last week of training and the first week of data collection. Staff from NSO and SC Malawi monitored data collection regularly throughout the data collection period.

### Data management and analysis

Data were double-entered into CSPro (United States Census Bureau, Washington DC, USA) by data entry clerks trained and supervised by NSO. Data supervisors ran CSPro quality checks cluster by cluster to identify discrepancies. Discrepancies were resolved by checking the paper questionnaire to determine the correct value. After data were cleaned, NSO removed all direct identifiers and shared the data set for analysis. Data were analyzed using Stata IC 14.2 (StataCorp; USA, College Station, Texas, USA). Frequencies and 95% confidence intervals were calculated for the household survey and HSA indicators. Household survey indicators included coverage and content of pregnancy and postnatal home visits. HSA survey indicators included proportion of HSAs conducting pregnancy and postnatal home visits in the last three months before the survey, the mean number of visits made during this period, supervision coverage and availability of CBMNC supplies and equipment. Confidence intervals for household survey indicators were adjusted for clustering. As the small sample size precluded multivariate analysis, we performed bivariate analysis to assess the association of selected covariates on coverage of postnatal home visits. Covariates assessed included pregnancy home visits (yes or no), HSA birth notification (yes or no), maternal education (Less than Form 3; Form 3 or higher) and maternal age (<25 years; 25 years or older) and were selected based on previous studies and considering sample size limitations [18]. Associations with *P*-values of <0.05 were considered statistically significant.

### Ethical considerations

The survey received ethical approval from the National Health Sciences Research Committee (NHSRC # 16/7/1617) of the MOH in Malawi, the ICF International Institutional Review Board and SC’s Ethics Review Committee. All participants provided informed oral consent, which interviewers documented on the survey tools.

## RESULTS

### Household survey findings

Interviews were completed with 140 mothers of children under six months of age. **Table 1** provides an overview of characteristics of mothers and babies included in the survey. The mean age of mothers was 26.1 years (standard deviation SD: 6.7; range 17-43 years); 23.6% of mothers were under 20 years of age and 12.1% were older than 35. Most mothers had attended at least primary level education, but only 17.9% had reached secondary or higher education. The child’s mean age was 2.8 months, and 35.0% of the sample was under two months of age. Most mothers (77.1%; 95% CI = 68.7-83.8) reported receiving at least one ANC visit from a medically skilled provider and nearly all women (97.9%; 95% CI = 93.5-99.3) reported delivering at a health facility.

**Table 1:** Background characteristics of mothers and babies included in the household survey (N = 140)

Background characteristics	Number	Percent
**Mothers**		
**Age (years):**		
<20	33	23.6
<20-24	39	27.9
≥25 y and older	68	48.6
**Highest level school attended:**		
None	11	7.9
Primary	104	74.3
Secondary or higher	25	17.9
**Highest level completed education:**		
Level 3 or less	98	70.0
Higher than level 3	42	30.0
**Babies**		
**Age (months):**		
<2	49	35.0
2-5	91	65.0
**Sex:**		
Male	78	55.7
Female	62	44.3

**Coverage and content of home visits:** Less than one-third of mothers reported receiving one or more home visits by an HSA during pregnancy and one-fifth received the recommended two or more visits (**Table 1**). Most visits took place after the first trimester. Among those receiving a home visit, 38.1% (95% CI = 23.3-55.5) reported that their husband/partner was present for at least one of the home visits. Most women (86.0%; 95% CI = 65.5-95.2) who received a pregnancy home visit reported receiving at least two priority messages. The most commonly cited areas of counselling (unprompted) from HSAs included maternal nutrition during pregnancy, facility delivery, danger signs during pregnancy, and birth preparations (emergency saving, emergency transport, clothing for baby, etc). About 83.7% (95% CI = 63.5-93.8) of mothers who received a pregnancy home visit indicated the HSA counselled them to inform him or her of the birth as soon as possible after delivery.

An estimated 20.7% (95%CI = 12.7-32.0) of mothers and newborns received a postnatal home visit by an HSA within eight days of delivery and 11.4% (95% CI = 6.8-18.5) received a visit within three days (**Table 2**). Only 7.1% (95% CI = 3.5-13.9) received two or more home visits from an HSA within the first week. The majority of newborns and mothers who received a postnatal home visit within eight days received all of the priority actions that HSAs were trained to provide as part of the postnatal home visits.

**Table 2:** Coverage and content of HSAs home visits during pregnancy and within the first week of delivery

Indicator	Denominator	Result (%)	95% CI
**Pregnancy home visits coverage:**			
Woman received at least one home visit during pregnancy from HSA	140	30.7	21.7-41.5
Woman received two or more home visits during pregnancy from HSA	140	20.0	13.0-29.4
**Timing of first home visit during pregnancy:**			
First trimester (1-3 months)	43	14.0	–
Second trimester (4 to 6 months)	43	58.1	–
Third trimester (7 to 9 months)	43	27.9	–
**Pregnancy home visit content (among those receiving visit):**			
HSA counselled on at least two priority messages during pregnancy* (*antenatal care, danger signs, skilled/facility delivery, birth planning, immediate newborn care*)	43	86.0	65.6-95.2
HSA counselled woman to inform/him or her of delivery	43	83.7	63.5-93.8
**Postnatal home visits coverage:**			
Mothers/newborns received at least one home visit from an HSA within eight days of delivery	140	20.7	12.7-32.0
Mothers/newborns who received two or more home visits from an HSAs within eight days of delivery	140	7.1	3.5-13.9
Mothers/newborns who received at least one home visit from an HSA within three days of delivery	140	11.4	6.8-18.5
**Postnatal home visit content (among those receiving visit):**			
Newborn received all four recommended actions during HSA postnatal visit^†^ *(check cord, temperature, counsel on newborn danger signs, weigh baby)*	29	75.9	54.2-89.7
Mother received all three recommended actions during postnatal home visit^†^ *(counsel on maternal danger signs, observe breastfeeding, discuss family planning)*	29	86.2	66.6-95.1

HSA – Health Surveillance Assistant, CI – confidence interval

*Question was unprompted.

†Question was prompted for each action.

### Factors associated with postnatal home visits coverage

**Table 3** presents the results of a bivariate analysis to explore the association between pregnancy home visits, HSA birth notification, maternal age and maternal education on receipt of a postnatal home visit within eight days after delivery. Women who received a home visit during pregnancy and women who reported the HSA had been informed of the birth were significantly more likely to receive a postnatal home visit in the first week of delivery. About 42.9% (95% CI = 33.4-52.8) of mothers reported that the HSA had been informed of the birth. Birth notification was significantly higher among those who received a pregnancy home visit than those who did not (74.4% vs 28.9%; *P* = 0.00). Communication to the HSA about the birth was in most cases (76.7%) made by an immediate family member (husband, mother, mother-in law) or other family member visiting the HSA in person or by the community action group (20.0%), with only 3.3% connections made by facility staff. Coverage of postnatal home visits among those who informed the HSA after delivery was 46.7% compared to 1.3% among those in which the HSA was not informed. Coverage of postnatal home visits did not vary significantly with maternal age or education level.

**Table 3:** Bivariate analysis of the association between coverage of a postnatal home visit within eight days after delivery and selected covariates

Variable	Denominator	Received postnatal home visit within 8 d (%)	*P*-value
**Received pregnancy home visit:**			
Yes	43	44.2	0.00
No	97	10.3	
**Informed HSA of birth:**			
Yes	60	46.7	0.00
No	80	1.3	
**Maternal education (highest completed):**			
Level 3 or less	98	21.4	0.75
Higher than Level 3	42	19.1	
**Maternal age (years):**			
<25	72	23.5	0.42
25 or older	68	18.1	

HSA – Health Surveillance Assistant

### HSA survey findings:

Interviews were completed with 33 HSAs from Ntcheu district, of which 31 had completed CBMNC training and were administered the full questionnaire. Most of the sampled HSAs were male (71.0%) and they ranged in age from 30 to 59 years, with close to two-thirds under the age of 40 (**Table 4**). While nearly all (96.8%) HSAs had completed Form 2 education, just over half had obtained their Malawi School Certificate of Education (Form 4). Three-quarters of HSAs resided in their catchment areas. The main modes of transportation were bicycles (65%) or walking (23%). Just over half of the HSAs (54.8%) reported it took less than one hour to reach the nearest health facility and 35.5% took between one and two hours and 9.7% took two hours or more. For those not living in their catchment areas, 74.2% reported they could reach their village clinic within 30 minutes.

**Table 4:** Background characteristics of HSAs trained in CBMNC (N = 31)

Background characteristics	Number	Percent
**Age:**		
<40 y	20	64.5
40 y or older	11	35.5
***Sex:***		
Male	22	71.0
Female	9	29.0
**Highest level of education:**		
Less than Form 4	14	45.2
Form 4 or higher (Malawi School Certificate of Education)	17	54.8
**Resident in catchment area:**	**23**	**74.2**
**Travel time from village to health facility:**		
Less than 30 min	8	25.8
30 min to less than one hour	9	29.0
One hour to less than two hours	11	35.5
Two or more hours	3	9.7

HSA – health surveillance assistant; CBMNC – community-based maternal newborn care

### CBMNC activity levels and functionality

Register reviews revealed that 83.9% (95% CI = 65.4-93.5) of HSAs had conducted at least one pregnancy home visit and 77.4% (95% CI = 58.4-89.3) had conducted one or more postnatal home visits in the three months before the survey (corresponding to the period of May to June 2016) (**Table 5**). On average, HSAs conducted 7.9 pregnancy home visits and 4.7 postnatal home visits over the three-month period (total of 12.6 home visits). HSAs resident in their communities conducted more home visits on average (13.1 compared to 11.3), however the difference was not statistically significant due to the small sample size. Overall, 83.9% (95% CI:65.4-93.5) of HSAs were considered ‘functional’ for CBMNC, with evidence of conducting pregnancy or post-natal home visits in the past three months according to register review and submitting a report on CBMNC activities in the past month.

**Table 5:** HSA pregnancy and postnatal home visit activity levels and supports for CMBNC (N = 31)

Indicator	Result	95% CI
**HSA activity levels (based on register review over last three months):**		
Pregnancy home visits:		
Percent of HSAs who conducted at least one pregnancy home visit in last three months	83.9	65.4-93.5
Percent of HSAs who conducted at least one follow-up pregnancy home visit in last three months	64.5	45.5-79.9
*Mean number of pregnancy home visits*	*7.9*	*5.6-10.3*
*Mean number of pregnancy follow-up visits*	*2.3*	*1.3-3.3*
Postnatal home visits:		
Percent of HSAs who conducted at least one postnatal home visit in last three months	77.4	*58.4-89.3*
Percent of HSAs who conducted at least one follow-up postnatal home visit in last three months	45.2	28.0-63.5
*Mean number of postnatal home visits*	*4.7*	*2.6-6.9*
*Mean number of postnatal follow-up visits*	*1.9*	*0.63-3.1*
Home visits overall:		
Percent of HSAs who conducted at least one home visit (pregnancy or postnatal) in the last three months	87.1	68.9-95.4
*Mean number of home visits (pregnancy and postnatal)*	*12.6*	*9.0-16.3*
**Levels of supervision and material support for CBMNC:**		
Supervision:		
Percent of HSAs who received at least one supervision visit for newborn health in the last three months	12.9	4.6-31.1
Equipment and supplies:		
Percent of HSAs with all four essential newborn commodities for CBMNC available and functional on day of assessment *(scale, thermometer, timer and counseling materials)*	83.9	65.4-93.5
Percent of HSAs with all CBMNC supplies and equipment available and functional on day of assessment *(all above plus register, CMBNC manual, referral slips and pregnancy listing notebook)*	16.1	2.4-29.8

HSA – health surveillance assistant, CBMNC – community-based maternal newborn care. CI – confidence interval

### Supervision, support and birth notification

Only a small number of HSAs (12.9%; 95% CI = 4.6-31.1) reported at least one supervision visit specific to newborn health in the past three months (**Table 5**). The supervision visits were conducted by senior HSAs (2/4) and SC staff (2/4). An estimated 83.9% (95% CI = 95% CI = 65.4-93.5) of HSAs were observed to have the essential newborn commodities for CBMNC (scale, thermometer, timer and counseling materials) at the time of the interview, but only 16.1% (95% CI = 2.4-29.8) had all eight items assessed. Newborn referral slips and exercise books for pregnancy listing had the lowest availability (29.0% and 54.8% respectively). Nearly all HSAs (96.8%; 95% CI = 78.4-99.6) reported being notified of births and three-quarters (74.2%; 95% CI = 55.1-87.1) indicated they were notified of the birth within three days. Family members (61.3%; 95% CI = 42.4%-77.3%), village health committees (35.8%; 95%: 20.1-54.5) and village leaders (19.4%; 8.5-38.1) were the most commonly mentioned sources of birth notification. Only 12.9% (95% CI = 4.6-31.1) of HSAs mentioned that health facility staff notified them – an important missed opportunity given that nearly all births occurred at health facilities.

## DISCUSSION

Our evaluation found low coverage of home visits by HSAs during pregnancy and the early postnatal period. Only about one-third of women received a home visit during pregnancy and few received the recommended two visits during pregnancy. Coverage of postnatal visits was even lower, with about one in five mothers and newborns receiving a visit within the first eight days (and less than one in 10 within three days of delivery). These low coverage levels are similar to those reported in the 2011 evaluation of the pilot CBMNC program in three districts (Thyolo, Dowa and Chitipa) [13,14,18]. Other similar findings included the importance of birth notification in facilitating postnatal visits and that women reached during pregnancy were more likely to receive postnatal home visits [18].

This evaluation also captured information from HSAs serving the clusters that were sampled in the household survey, which revealed that the majority of HSAs trained in CBMNC were well-equipped (with the exception of newborn referral slips) and most had documented evidence in their registers of providing pregnancy and postnatal home visits during the three months before the survey. However, activity levels were well below what would be required to achieve high coverage. Assuming the average HSA’s catchment population of 2000 and a crude birth rate of 42/1000, one would expect about 84 pregnancies/births per year [16]. According to the CBMNC schedule of two home visits during pregnancy and three home visits in the first eight days after delivery, each HSA would need to conduct a minimum of 168 pregnancy home visits and 252 postnatal home visits per year (420 total), translating into approximately 35 home visits per month or 105 per quarter. In contrast, HSAs were conducting about 12-13 home visits per quarter, which tracks quite well with the observed coverage levels. We found that HSAs who resided full-time in their communities conducted more home visits than those who lived elsewhere, however the results were not statistically significant due to the small sample size. The evaluation of the CBMNC pilot also reported higher CBMNC activity levels among resident HSAs [18]. As HSAs assigned to more remote, poorer communities are less likely to reside full-time at their site, this can bias coverage toward wealthier communities, despite the intent of community-based programs to help address inequities in access to health care [13]. While we were unable to assess coverage by socioeconomic status, earlier studies of CBMNC suggest that that households in the wealthiest quintile are slightly more likely than those in the poorer quintiles to receive home visits, in part because of the underlying disparities in access to a resident HSA [13].

Supervision of HSAs for the CBMNC component was very low. Only a handful of HSAs had received any supervision specific to the newborn package; these low levels could be due the senior HSAs only receiving formal supervision training for CBMNC in November 2016 (after the evaluation). Analysis of supervision coverage and supply chain supports for other community-based programs such as iCCM are mixed. The HSAs interviewed as part of the evaluation of the larger iCCM program implemented through RAcE reported much higher levels of supervision, with two-thirds of the HSAs reporting supervision for iCCM in the last three months [19]. However, other studies of program support for iCCM found lower levels of supervision (38% supervision coverage in last three months) and breaches in supply chain management, suggesting that sustaining consistent levels of program support for HSAs to deliver community-based activities remains a persistent challenge for the MOH and partners [20]. Going forward, it will be possible to conduct CBMNC supervision jointly with iCCM, although adjustments may be required as current supervision approach for CBMNC involves visiting the mothers in their own households and may not be feasible in most cases.

The household survey and HSAs findings taken together highlight the limited role health facilities are presently playing in helping to link HSAs to recently delivered mothers and their newborns. Given that nearly all births occurred at health facilities, this is a critical missed opportunity. Further efforts should be made to explore feasible options for facilities to notify HSAs of births in their catchment areas; it could be possible to have senior HSAs coordinate with the clinical staff involved in post-natal discharge to identify which HSAs serves the catchment area where the family is from before they leave to go home. At the community level, families and other community members may also be mobilized to help alert HSAs that a birth has taken place. Women who reported that an HSA was notified of the birth were more likely to receive a postnatal home visit; however, about half of these women still did not receive a postnatal home visit. This suggests that other barriers play an important role that needs to be further explored.

Despite revisions to the CBMNC package, including a somewhat reduced schedule of home visits, the performance of the CBMNC program appears to have reached a coverage plateau at a very low level, particularly for postnatal home visits. Even if efforts are successful in strengthening linkages between facilities and families and notification of HSAs is improved, it is unlikely that HSAs would be able to conduct home visits at the level required to achieve high coverage. A costing analysis found that it took an HSA 1.5 hours on average to conduct a CBMNC home visit, including time for preparation, travel and interaction with the family [14]. In addition to their role in CBMNC, HSAs are officially responsible for a wide range health and sanitation tasks at community and facility levels – estimated to be more than 250 [19,21]. These tasks include delivery of life-saving interventions such as supporting immunization outreach and providing assessment and treatment for childhood illness, and increased time spent on home visits could be a trade-off with other important health interventions. These struggles are not unique to Malawi, with many other countries failing to reach high coverage of home visits despite strong program support [11]. In addition, large scale evaluations of the coverage of other community-based interventions delivered through HSAs, such as iCCM, have also reported lower than expected coverage despite relatively strong implementation [22]. Given this reality, it is worth revisiting the feasibility of the CBMNC package in its current form and exploring options to focus on reaching those at greatest risk – such as first time mothers, women who have given birth to preterm or small babies, and women who deliver outside a health facility – and investigating opportunities to shift some components such as counselling to other community-based volunteers more likely to reside full-time in the community and have greater access to families [11,12]. Although the 2015-2016 DHS survey showed that more than 90% of deliveries in Malawi occurred at facilities and about three-quarters of mothers stayed in facility at least one to two days following delivery, just 44.6% of mothers who delivered at facility reported receiving a PNC check within two days [23]. For newborns, the results were similar, but slightly higher: 63.1% of babies born in facility received PNC within two days [23]. These findings imply that further efforts should be placed to improve the coverage and quality of these facility-based postnatal contacts with mothers and newborns [12]. Further investigations should be carried out to explore the potential for HSAs, who already spend considerable time at health facilities, to support facility staff to provide aspects of postnatal care and counselling at health facilities as well as in the community.

### Limitations

Our evaluation had some important limitations. The CBMNC program had been implemented for a short period of time, with most HSAs completing training nine to 12 months prior to the survey, but a small number of HSAs were trained only five months prior to the survey. Although the design restricted the sample to infants under six months of age to maximize potential exposure to CBMNC, some women in our sample may have completed their pregnancy before HSAs were providing home visits, potentially underestimating coverage of home visits during pregnancy. Another limitation to the study is survival bias, since only mothers with a live birth were sampled for our study and women who experienced stillbirth or early infant death and may have had lower levels of care would have been excluded; however the effect of this would be fairly small and tend to bias the sample toward higher coverage (and coverage was found to be quite low). The sampling approach for the household survey defined clusters using census enumeration areas, which did not perfectly align with HSA catchment areas. This may have resulted in some clusters without access to a trained HSA. Indicators on coverage of postnatal contact and coverage relied on maternal recall using a complex series of questions similar to the DHS and MICS survey questionnaires on postnatal care and thus are subject to recall bias and measurement error [24]. However, studies show that most mothers are able to recall interactions with health providers and specific interventions received if the questions are adequately prompted and the actions notable (eg, required asking a question or use of equipment) [25]. Additionally, the small sample size in the household survey resulted in relatively large confidence intervals around some estimates and limited our ability to conduct multivariate analysis of factors associated with coverage of postnatal home visits or assess equity. Similarly, the small number of HSAs interviewed precluded disaggregated analysis to better understand factors associated with HSA activity levels and we did not capture information on workload or how HSAs spent their time. Finally, the evaluation was conducted in a single district of Malawi, and an evaluation of the previous CBMNC package in multiple districts found considerable variation in coverage of home visits and as such it unknown how generalizable the current results are to other districts implementing CBMNC interventions [14]. As the revised CBMNC package has continued to be rolled out across Malawi, further studies covering multiple districts and employing mixed methods could provide valuable information on factors driving performance and inform community and facility-based strategies to optimize care for mothers and babies in Malawi.

## CONCLUSIONS

We found low coverage of home visits by HSAs during pregnancy and soon after delivery in a well-supported program delivery environment. Most HSAs were conducting home visits, but not at the level needed to reach high coverage. These findings were similar to previous studies, calling into question the feasibility of the current visitation schedule in the CBMNC package. It is time to re-align the CBMNC package with what the existing platform can deliver and identify strategies to support HSAs to implement home visits. Options for exploration include targeting home visits to those at greatest risk (first time mothers, women who have previously given birth preterm babies, women who deliver outside facility), supporting health facility staff to improve birth notification and engaging communities to increase demand and define locally appropriate solutions. Targeted efforts are also needed to improve the coverage and quality of essential interventions through the existing high level of facility-based prenatal and postnatal contacts with women and their babies in Malawi.

## Additional Material

Online Supplementary Document
