# Supplementary material for: Home visits by community health workers for pregnant mothers and newborns: coverage plateau in Malawi
Source: J Glob Health. 2019 Jun 25;9(1):010808. doi: 10.7189/jogh.09.010808 (PMC6596344; doi:10.7189/jogh.09.010808)
Supplement: Online Supplementary Document [file jogh-09-010808-s001.pdf]

J Glob Health 2019; 9: 010808

Online Supplementary Document

Table S1. Health Surveillance Assistant (HSA) Tasks for Community Based Maternal Newborn Care (CBMNC) in Malawi

1. ***Identify pregnant women in the community*** *so that HSA can make home visits during pregnancy and in the first days after birth for the greatest impact*

2. ***Make two home visits to all pregnant women*** *in the community:*

   First pregnancy visit *— as early in pregnancy as possible —*
   - *To encourage the pregnant woman to go for early antenatal care*
   - *To encourage her to learn her HIV status so that she can take action to best protect her health and protect her baby from HIV*
   - *To promote birth in a health facility*
   - *To help prepare for birth*
   - *To teach home care for the pregnant woman.*

   Second pregnancy visit *— about 2 months before delivery —*
   - *To review antenatal care visits, plans for birth, and home care for the pregnant woman.*
   - *To encourage the family to follow optimal newborn care practices mmediately after birth.*
   - *If the woman has HIV, to check that she is taking ARVs every day and will continue through delivery and breastfeeding.*

3. ***Make 3 home visits after birth*** *for all mothers and babies, regardless of place of birth.*

   First postnatal visit *— on Day 1 after birth —*
   - *To assess the mother and baby for signs of illness, weigh the baby, and help the mother with early and exclusive breastfeeding and keeping the baby warm.*
   - *If the mother has HIV, to check that the mother is taking her ARVs and giving the baby the ARV syrup at the same time every day.*

   Second postnatal visit — *on Day 3 after birth —*
   - *To assess for signs of illness, help the mother to sustain breastfeeding and prevent breastfeeding problems, and advise on optimal care for the*

mother and her baby.

- *If the mother has HIV, to check that the mother is taking her ARVs and giving the baby ARV syrup at the same time every day. (NOTE: If the first postnatal visit is delayed until day 2 for some reason, this visit should still be made on day 3.)*

Postnatal visit 3 — *on Day 8 after birth* —

- *To assess for signs of illness, and advise on optimal care beyond the first week of life.*
- *If the mother has HIV, to encourage her to continue taking her ARVs and giving the baby the ARV syrup at the same time every day; and to bring the baby to the health facility when the baby is 4–6 weeks old, so that the baby can be tested for HIV and started on cotrimoxazole.*
  (NOTE: If the second postnatal visit is delayed for some reason, this visit should still be made on day 8.)

4. ***Make two extra home visits after birth for small babies (birth weight less than 2.5 kg) — on day 2 and day 14 —*** *so that the HSA can provide the extra care that small babies need*

5. ***Make a follow-up visit for a baby who is referred to a health facility for illness***

Source: Malawi MOH. Caring for the Newborn at Home: Participant Manual. Lilongwe, 2014
